# Sebaceous breast carcinoma

**DOI:** 10.4322/acr.2021.365

**Published:** 2022-04-14

**Authors:** Natália Nobre de Alencar, Diego Agra de Souza, Alexandre Alves Lourenço, Raimunda Ribeiro da Silva

**Affiliations:** 1 Universidade Federal do Maranhão (UFMA), Hospital Universitário, São Luís, MA, Brasil; 2 Hospital do Câncer Aldenora Bello, Laboratório de Patologia, São Luís, MA, Brasil

**Keywords:** Adenocarcinoma, Adenocarcinoma Sebaceous, Breast Neoplasms, Carcinoma, Sebaceous Gland Neoplasms

## Abstract

Breast sebaceous carcinoma is one of the rarest breast neoplasms, with less than 30 cases reported worldwide. Due to the rarity, the new WHO classification of breast tumors grouped these tumors among the ductal carcinoma. A detailed description of these cases is relevant due to the insufficient knowledge about the prognosis of this neoplasm. We report the clinical, histological, and immunohistochemical characteristics of a case of sebaceous carcinoma of the breast in an 81-year-old woman with a right breast nodule. The tumor was composed of nests of a varying mixture of sebaceous cells with abundant slightly vacuolated cytoplasm, surrounded by smaller oval-to-fusiform cells with eosinophilic cytoplasm without vacuolization. No lymph node metastases were present. The immunohistochemical reactions were positive for GATA3, EMA, CD15, and GCDFP15 (focal staining), and negative for RE, RP, and HER-2. The tumor was classified as triple-negative. Morphologically, the differential diagnoses included skin sebaceous carcinoma, lipid-rich carcinoma, apocrine carcinoma, and glycogen-rich clear cell carcinoma. Most of the previously reported cases were positive for RE and RP, which generally was associated with a better prognosis. However, some cases presented a more aggressive behavior with distant and lymph node metastases.

## INTRODUCTION

The breast sebaceous carcinoma (SC) is a rare primary carcinoma with prominent sebaceous differentiation but without evidence of derivation from cutaneous adnexa.^1^^,^^2^ Only a few cases have been reported; thus, the knowledge about the pathophysiology and prognosis is limited.^3^ We report a case of breast sebaceous carcinoma and review the clinical, immunohistochemical, and histopathologic findings of previously reported cases.

A literature review was performed using the descriptors “sebaceous”, “breast” and “carcinoma”. The following search platforms were used: Embase, Scielo, BVS, Pubmed, Google Scholar. Initially, 404 articles were found in Google Scholar, 257 in Embase, 1 in the BVS, and none in SciElo. Articles related to genetic research or description of sebaceous tumors from other locations were excluded. Due to the scant information, abstracts of case reports presented at congresses were also excluded. Some articles were indexed in two or more databases. Most of the selected articles were published in English, except for two of them. These were published in German and Spanish. Finally, 25 well-described and well-documented case reports articles were selected. These articles contain reports of 27 cases of sebaceous breast carcinoma.

## CASE REPORT

An 81-year-old black woman sought medical care after self-detection of a lump in her right breast. She denied alcoholism and smoking. History of hysterectomy for leiomyomatosis. Upon clinical examination, a 5.0 cm nodule was noted in the superomedial quadrant of the right breast. She underwent a biopsy five months later. Histopathological examination of the biopsy specimen revealed histological Nottingham grade II invasive ductal carcinoma of the breast. The delayed diagnosis was due to the patient’s social and financial restraints.

Four months after the biopsy, she was referred to our hospital. The patient underwent segmental resection of the breast and sentinel lymph node and started adjuvant radiotherapy a month later. The analysis of the tissue sections showed a brownish firm-elastic tumor with well-defined margins, measuring 3.4 cm in its longest axis, and at a distance of 0.5 cm from the skin.

**Figure 1:**
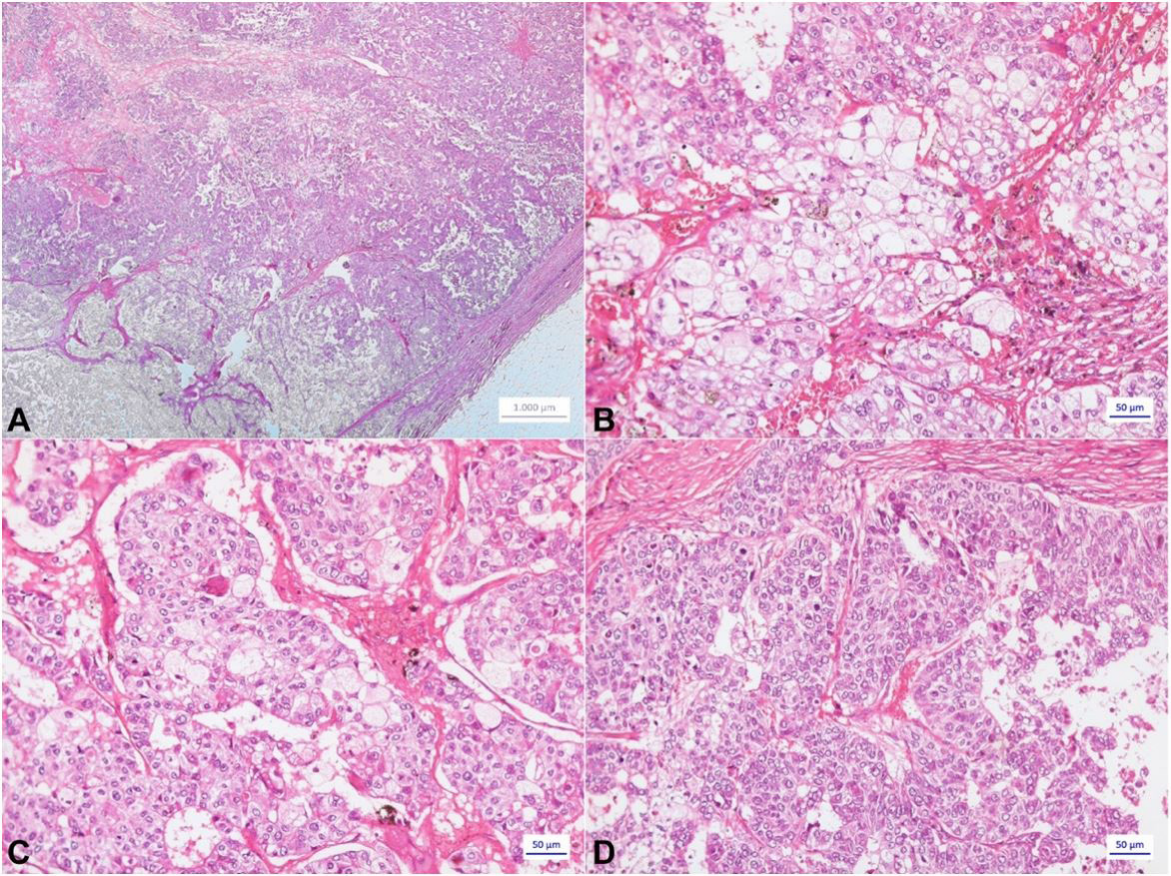
Photomicrograph of the tumor. **A**, **B**, **C** – Lobules of tumor cells with abundant vacuolated cytoplasm, surrounded by more basophilic tumor cells in the periphery of the lobules (HE); **D** – Clusters of cells without evidence of sebaceous differentiation, indistinguishable from a classic ductal carcinoma (HE).

Histopathological examination revealed breast sebaceous carcinoma of Bloom and Richardson^4^ grade III type (nuclear grade 3, histologic grade 3, mitotic grade 3) (Figure 1). A desmoplastic reaction was observed with mild inflammatory infiltrates, absence of angiolymphatic and perineural invasion, as well as the absence of metastatic disease in the examined sentinel lymph node. The pathologic staging was pT2 pN0 (TNM 8th edition).

**Figure 2:**
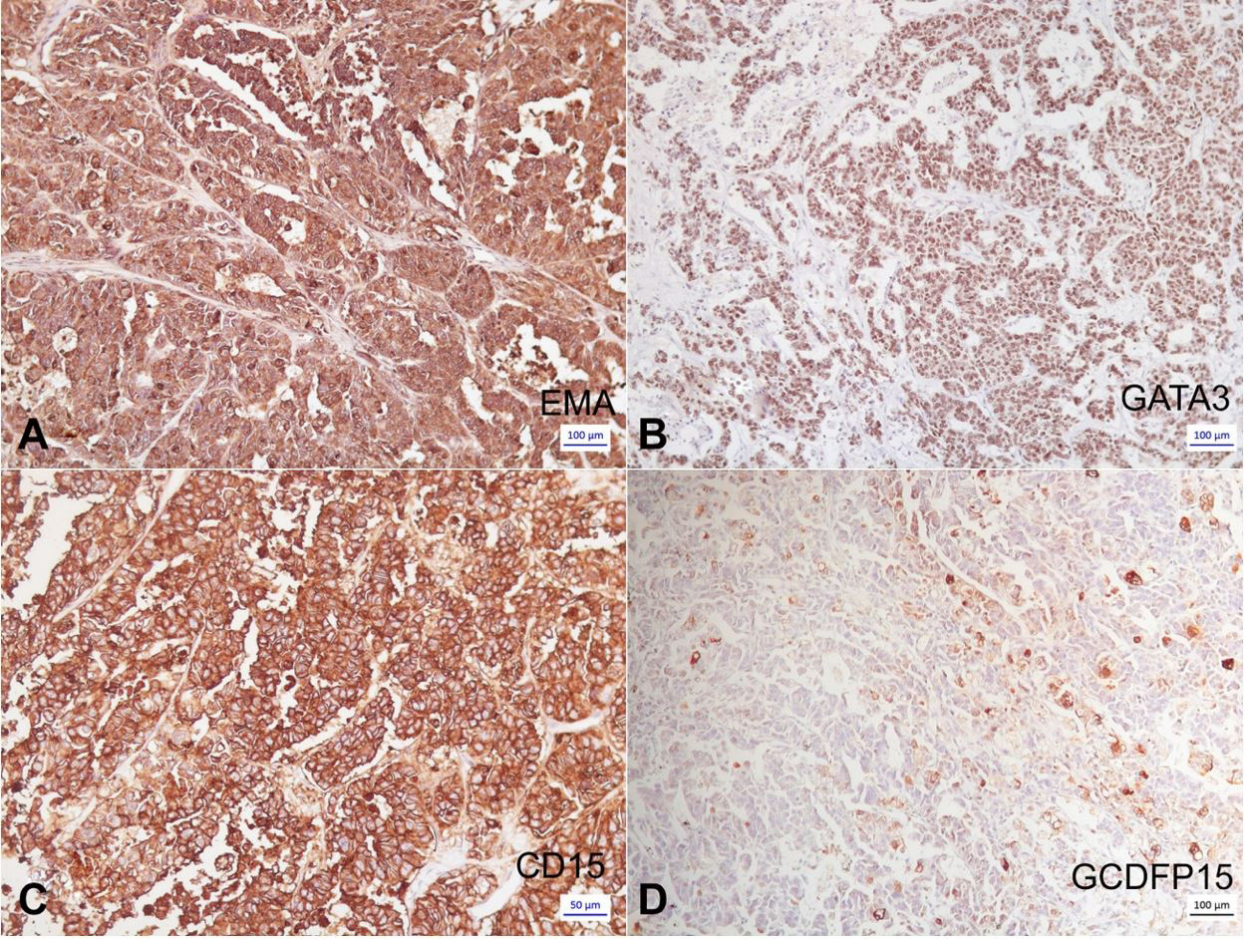
Photomicrograph of the tumor. Immunohistochemical panel. **A** – Positive expression of EMA in the tumor cells (100 x); **B** – Positive expression of GATA-3 in the tumor cells (25 x); **C** – Strongly positive expression of CD15 in the tumor cells with sebaceous differentiation (100 x); **D** – Focal positivity of GCDFP15 in the tumor Cells (25X)

The immunohistochemical panel revealed positivity for EMA (Figure 2A), GATA3 (Figure 2B), AE1AE3, CK7, CD15 (Figure 2C), GCDFP15 (focal staining) (Figure 2D), PMS2, MLH1, MSH2, MSH6, and negativity for P63, Melan-A, HMB45, 1A4, estrogen receptor (ER), progesterone receptor (RP), mammaglobin, calponin, and HER2 (+1). Thus, the tumor was classified as triple-negative sebaceous carcinoma (SC) of the breast.

The risks and benefits of an 81-year-old woman with other comorbidities were considered, and it was decided not to undergo adjuvant chemotherapy. Follow-up was conducted every three months for nine months.

## DISCUSSION

Primary breast SC is rare and resembles skin sebaceous carcinoma. According to the current WHO classification, primary breast SC shows prominent sebaceous differentiation and no evidence of derivation from cutaneous adnexa. The previous WHO classification included the criterion that sebaceous differentiation occurs in at least 50% of cells.^1^^,^^2^

Histologically, it is characterized by a lobulated or nested proliferation of a varying mixture of sebaceous cells with abundant, slightly vacuolated cytoplasm surrounded by smaller, oval to fusiform cells with eosinophilic cytoplasm without vacuolization. The nuclei of the sebaceous component are mainly located eccentrically and vary from small monomorphic cells to larger pleomorphic cells; mitotic figures may be abundant.^2^ This was observed in our case (Figure 1).

The differential diagnosis of breast SC includes skin sebaceous carcinoma, lipid-rich carcinoma, apocrine carcinoma, and glycogen-rich clear cell carcinoma.^1^^,^^3^^,^^5^^-^7

The distinction of breast SC from skin SC can be made when there is no microscopic or macroscopic connection between the tumor and the overlying skin or when the tumor is surrounded by breast tissue.^3^^,^^8^ In our case, there were no signs of skin involvement. The main characteristics of the other differential diagnoses are summarized in Table 1.

**Table 1:** Summary of the main morphological differences between breast SC (sebaceous carcinoma), skin SC, lipid-rich carcinoma, and glycogen-rich clear cell carcinoma

	**Breast SC**	**Skin SC**	**Lipid-rich carcinoma**	**Apocrine carcinoma**	**Glycogen-rich clear cell carcinoma**
**Cell types**	2 types	2 types	+2 types	2 types	1 type
**Cytoplasm**	Vacuolated	Vacuolated	Vacuolated, lipid-rich	Granular eosinophilic and/or vacuolated	Clear and abundant, glycogen-rich
**Architecture**	Lobules or nests	Lobules and nests	Irregular	Glandular and tubular	Solid or papillary
**Mitoses**	Present (moderate to severe)	Present	Present	Present	Present
		(Moderate to severe)	(Moderate to severe)	(Moderate to severe)	
**Connection with the skin**	Absent	Absent	Absent	Absent	Absent
**GCDFP-15**	Generally -			+	
**PAS**	-	-	-	-	+

PAS – Ácido periódico de Schiff.

The origin of sebaceous cells in breast carcinoma is still unclear. The histogenic hypotheses include the existence of a ductal reserve cell capable of sebaceous differentiation and the displacement of an embryonic group of epidermal cells in the breast parenchyma.^5^

To date, 27 well-documented cases have been reported in English, including ours. The clinical and pathologic characteristics observed in these cases are available in Table 2. Out of the cases in these reports, twenty-three patients were women with ages within 25 to 85 years and three were men aged between 55 and 70 years, the mean age being 60,3 years (53,08- 67,52, IC 95%). Our patient’s age was within the expected age range.

**Table 2:** Clinicopathological summary and follow-up of patients with breast sebaceous carcinoma

**Ref.**	**Age/Sex**	**Size**	**LN metastases**	**FOLLOW UP**
9	74/F	5.0 cm		Alive without evidence of disease for 6 months after surgery.
10	55/M	5.0 cm	-	Alive without evidence of disease for 10 months after surgery.
5	45/F	2.5 cm	-	Alive with disease for 96 months after surgery.
				Skin and bone metastases.
11	46/F	1.4 cm		Alive without evidence of disease for 6 months after surgery.
12	63/F	2 cm	+	
13	50/F	2 cm	+	Alive without evidence of disease for 24 months after surgery.
14	85/F	2.5 cm		
				Disease recurrence after 10 months after surgery
3	65/F	1.6 cm	+	Alive without evidence of disease for 27 months after surgery.
3	61/F	1.7 cm	+	Liver, lung, and bone metastases, pathologic fracture of the femur, arrhythmia.
				Deceased from the disease 28 months after surgery.
3	66/F	3 cm	+	Endometrial carcinoma; metastases to the mediastinal and supraclavicular lymph nodes.
				Alive with disease for 70 months after surgery.
3	25/F		-	Had a child.
				Alive without evidence of disease for 75 months after surgery.
15	80/F	3.5 cm	-	Alive with evidence of disease.
				Metastatic cervical carcinoma 16 months after surgery.
6	55/F	6.5 cm	-	Alive without evidence of disease for 13 months after surgery.
6	42/F	2.5 cm	+	Alive without evidence of disease for 15 months after surgery.
16	74/F	2.3 cm	-	Alive without evidence of disease for 58 months after surgery.
17	51/F	2.0 cm	-	
Our case	81/F	3.4 cm		Alive without evidence of disease for 9 months after surgery
18	71/F	1.4 cm	-	Alive without evidence of disease for 18 months after surgery.
7	70/M	1.7 cm	-	
19	47/F	1.3 cm	-	Alive without evidence of disease for 16 months after surgery.
20	79/F	1.5 cm		Alive without evidence of disease for 36 months after surgery
21	63/F	1.8 cm	+	
22	65/F	1.1 cm	+	Refused further treatment. Recurrence disease 4 months after surgery. Died at ninth month of follow-up.
22	71/F	2.7 cm	-	Alive without evidence of disease for 100 months after surgery.
23	70/M	1.7 cm	-	Alive without evidence of disease for 2 months after surgery.
24	65/F	3.0 cm	+	Alive without evidence of disease for 4 months after surgery.
25	53/F	1.2 cm	-	

LN= lymph nodes; F = Sexo feminino; M = Sexo masculino; - = Negativo para metastases em linfonodo sentinela; + = Positivo para metátastases em linfonodo sentinela.

It was also observed that the mean size of the lesions was 2,7 cm (2,03-3,38, IC 95%). Only 22 cases had reported the prognoses: nine of the 23 patients had metastases to the axillary lymph node, four patients experienced an aggressive behavior and distant metastasis (Table 2).

As shown in Table 3, immunohistochemical characterization (positive reactions) was: ER (+ 16/23, 69,57% IC 95%: 0.4894 to 0.8459), PR (+ 13/17, 76,47% IC 95%: 0.5223 to 0.9095), HER-2 (+2/21, 9,52% IC 95%: 0.0145 to 0.3012), EMA (+11/12, 91,67% IC 95% 0.6247 a >0.9999), and GCDFP-15 (+1/8, 12,5% IC 95% 0.0011 to 0.4922). Thus, breast sebaceous carcinoma was shown to have a high positive expression of ER, PR and EMA (Figure 2A) and a low positive expression of HER-2 and GCDFP-15 (Figure 2D).

**Table 3:** Summary of immunohistochemical data for breast sebaceous carcinoma

**Ref**	**ER**	**PR**	**HER2**	**Ki67**	**P53**	**GCDFP-15**	**EMA**	**AR**	**GATA3**	**Mglb**	**CD15**	**AE1AE3**	**S**
9							+					+	
5	+	+	-	16%									
12	+	+	-	38%			+	-					
13	-	-	+	30%			+	+				+	
14	-	-	-	25%		-						+	
3	+	+	-	30%	+		+						
	-	-	-	80%	-		+						
	+	+	-	5%		-	-						
	+	+	-			-	+	-					
15	-	-	-				+	-					
6	-	-	+	50%		-							
6	+	-	-	60%	+	-							
OC	-	-	-	25%	-	+ f	+		+	-	+	+	
19	-	-	-	90%									
7	+	+	-	20%				+		+ (focal)			
17	+	+f	-	20%				+					
16	+	+	-	20%				+	+				
18	+	+f	-	35%			+	+					
20	+	+	-				+					+	
21	+			60%		-			+				
22	-	-	-					+f	+				
22	+	-						-	+				
23	+	+		20%				+	+	+f			
24	+	+	-	60%		-	+					+	+
25	+	+	-	40%				-	+				+f

ER= Estrogen-receptor; +f = focal, Mgbl= mammaglobin; OC= our case; PR=progesterone receptor, Ref= reference; S= synaptophysin; AR = androgen receptor; EMA = epithelial membrane antingen; + = Positive.

The high ER and PR positivity rate in breast sebaceous carcinoma indicates that it is a hormone receptor-dependent breast cancer.^5^ Based on hormone receptor state and HER2 expression, the number of cases of sebaceous breast cancer of the luminal, HER2-positive, and triple-negative subtypes were thirteen, two, and five, respectively, which indicates that the luminal type is the most common subtype of breast SC and that this disease has a good prognosis and low invasiveness.^5^ However, some cases exhibited a more aggressive lineage, with distant and lymph node metastases.^3^^,^^5^^,^^6^^,^^10^^,^^12^^,^^13^ Our patient, unlike the majority of the described cases, was negative for ER, PR, and HER-2, and was therefore classified as triple-negative.

Positivity for GATA3 (Figure 2B) and focal positivity for GCDFP15 (Figure 2D) reinforced the breast origin of the tumor cells, and positivity for CD15 (Figure 2C) confirmed sebaceous differentiation. Our case was the first described in the literature for which CD15 immunostaining was performed; this marker was shown to be positive in skin sebaceous carcinomas.^26^^,^^27^

We understand as the first description of sebaceous breast carcinoma the publication by Prescott RJ^9^ of 1992. However, Bogaert and Madalgue^25^ publication is cited as the first description. We find that the latter fits better the diagnosis of lipid-rich carcinoma due to sebaceous carcinoma.^25^

Finally, the positive expression of the PMS2, MLH1, MSH2, and MSH6 proteins, used to detect microsatellite instability, permitted us to rule out the Muir-Torres syndrome, which is characterized by the coexistence of sebaceous gland neoplasms and multiple visceral neoplasms.^8^

## CONCLUSION

We report a rare case of an 81-year-old woman with breast sebaceous carcinoma who, unlike what is expected for this subtype, was negative for RE, RP, and HER2. The tumor was thus classified as triple-negative, while most other reported cases had a high rate of positive expression of RE and RP and a low rate of positive expression of HER-2 and GCDFP-15. Our case is the first to be described in the literature, which had CD15 immunostaining performed, a positive marker in skin sebaceous carcinomas. Although breast sebaceous carcinomas are mostly of the luminal subtype, associated with a good prognosis, our analysis revealed cases that present a more aggressive lineage with distant and lymph node metastases. We conclude that the pathophysiology and prognosis of breast sebaceous carcinoma are poorly understood due to its rareness, and requires further study.
